# Risk factors of failure to achieve textbook outcome in patients after pancreatoduodenectomy: a systematic review and meta-analysis

**DOI:** 10.1097/JS9.0000000000002299

**Published:** 2025-02-24

**Authors:** Jiajun Yuan, Changjie Du, Hongyu Wu, Tao Zhong, Qilong Zhai, Jialun Peng, Nan Liu, Jinzheng Li

**Affiliations:** a Department of Hepatobiliary Surgery, The Second Affiliated Hospital of Chongqing Medical University, Chongqing, China; b Department of Hepatobiliary Surgery, The Affiliated Yongchuan Hospital of Chongqing Medical University, Yongchuan, Chongqing, China

**Keywords:** meta-analysis, pancreaticoduodenectomy, risk factors, textbook outcome

## Abstract

**Background:**

Pancreatoduodenectomy (PD) represents one of the most technically demanding surgical procedures, characterized by extensive surgical trauma and high perioperative morbidity. Single outcome measures are insufficient to comprehensively assess the surgical quality of PD. Textbook outcome (TO), as an integrated evaluation system incorporating multiple clinical parameters, offers an objective, reliable, and comprehensive assessment of surgical performance. This systematic review and meta-analysis aimed to identify risk factors associated with failure to achieve textbook outcome (non-TO) following pancreatoduodenectomy.

**Materials and methods:**

We systematically searched international databases (PubMed, Web of Science, EMBASE, and Cochrane Library) and Chinese databases (China National Knowledge Infrastructure, Weipu Chinese Journals Service Platform, Wanfang Data, and SinoMed) for studies on risk factors of failure to achieve textbook outcome after pancreatoduodenectomy from inception to 31 December 2024. Pooled odds ratios (ORs) with 95% confidence intervals (CIs) were calculated using random-effects or fixed-effects models. Heterogeneity testing, sensitivity analysis, and publication bias assessment were conducted.

**Results:**

Ten studies involving 5238 patients were included in this meta-analysis. Among the 18 factors evaluated, five were significantly associated with failure to achieve textbook outcome after pancreatoduodenectomy: preoperative biliary drainage (OR = 2.09, 95%CI [1.30-3.36], *P* = 0.002), smaller tumor size (OR = 1.36, 95%CI [1.02-1.81], *P* = 0.04), soft pancreatic texture (OR = 2.25, 95%CI [1.01-5.02], *P* = 0.05), small pancreatic duct diameter (OR = 2.30, 95%CI [1.62-3.28], *P* < 0.00001), and increased intraoperative blood loss (OR = 4.14, 95%CI [1.16-14.83], *P* = 0.03). The remaining 13 factors showed no significant association with failure to achieve textbook outcome.

**Conclusion:**

This meta-analysis identified preoperative biliary drainage, tumor morphological characteristics (including size and texture), pancreatic duct diameter, and intraoperative blood loss as key factors affecting the achievement of textbook outcome after pancreatoduodenectomy. These findings may help surgeons identify high-risk patients for failure to achieve textbook outcome, enabling personalized surgical strategies and optimized perioperative management to improve textbook outcome rates.

**Highlights**

- First meta-analysis evaluating risk factors of failure to achieve textbook outcome after pancreatoduodenectomy.
- Five significant predictors were identified through comprehensive analysis of published literature.
- Preoperative biliary drainage and soft pancreatic texture increase risk of non-textbook outcome.
- Small pancreatic duct and increased blood loss are associated with worse surgical outcomes.
- Findings provide guidance for risk assessment and surgical decision-making.

## Introduction

Pancreatoduodenectomy (PD) is the standard surgical procedure for treating tumors of the pancreatic head, distal common bile duct, and ampulla of Vater^[1]^. First performed by Italian surgeon Alessandro Codivila in 1898^[2]^ and subsequently modified by Allen Whipple in the 1930s^[3]^, this procedure has evolved significantly over the past century. Despite its maturity, PD remains challenging due to complex regional anatomy, extensive resection requirements, and difficult digestive tract reconstruction. Postoperative complications such as intra-abdominal hemorrhage, pancreatic fistula, bile leakage, and abdominal infection are common and severe^[4]^, making quality control particularly crucial. Traditionally, surgical quality assessment relied on single outcome measures, including mortality rate, major complication rate, operation time, length of hospital stay, and readmission rate. However, this approach neither comprehensively reflects the overall surgical quality nor accurately evaluates the actual differences between healthcare institutions^[5]^.

The concept of Textbook Outcome (TO) was first introduced by Dutch colorectal surgeons in 2013 as a comprehensive quality assessment standard for surgical procedures^[6]^. Since its inception, TO has been widely adopted for quality assessment across various surgical disciplines^[7-9]^. In 2020, the Dutch Pancreatic Cancer Group (DPCG) formally established TO criteria for pancreatic surgery based on international expert consensus. These criteria specify that patients should experience no in-hospital mortality, no Clavien-Dindo grade III or higher complications, no clinically relevant pancreatic fistula, no postoperative hemorrhage, no bile leakage, and no readmission within 30 days^[10]^. Compared to traditional single outcome measures, TO provides a more comprehensive, accurate, and reliable assessment of short-term outcomes and overall surgical quality^[11]^.

Since 2020, several studies have investigated independent factors affecting the achievement of textbook outcome after pancreatoduodenectomy, including gender, pancreatic texture, main pancreatic duct diameter, intraoperative blood loss, and operation time. However, these studies often reached inconsistent conclusions and were limited by small sample sizes. Therefore, we conducted a comprehensive meta-analysis of all relevant Chinese and English literature published since the establishment of TO criteria for pancreatic surgery in 2020. This analysis included 10 studies with 5238 patients and evaluated 18 potential risk factors, aiming to identify risk factors of failure to achieve textbook outcome after pancreatoduodenectomy.

## Materials and methods

### Search strategy

The study protocol was registered on PROSPERO, an international prospective register of systematic reviews. The implementation strictly followed the PRISMA guidelines^[12]^, and underwent quality assessment using the AMSTAR Checklist^[13]^. We searched four international databases (PubMed, Web of Science, Embase, and Cochrane Library) and four Chinese databases (China National Knowledge Infrastructure, Weipu Chinese Journals Service Platform, Wanfang Data, and SinoMed) through 31 December 2024. The search strategy combined MeSH terms and free text words. The PubMed search string was as follows: ((Pancreaticoduodenectomy [Mesh]) OR (Pancreaticoduodenectomies [Title/Abstract]) OR (Pancreatoduodenectomy [Title/Abstract]) OR (Pancreatoduodenectomies [Title/Abstract]) OR (Duodenopancreatectomy [Title/Abstract]) OR (Duodenopancreatectomies [Title/Abstract]) OR (Pancreatectomy [Mesh]) OR (Pancreatectomies [Title/Abstract]) OR (Whipple [Title/Abstract]) OR (KauschWhipple [Title/Abstract]) OR (pp Whipple [Title/Abstract]) OR (Pancreatic head resection [Title/Abstract]) OR (Pancreatic Neoplasms/surgery [Title/Abstract]) OR (PD [Title/Abstract]) OR (PPPD [Title/Abstract]) OR (Pancreatectomy [Title/Abstract]) AND (((textbook [Title/Abstract]) OR (textbook outcome [Title/Abstract]) OR (textbook outcomes [Title/Abstract])). Detailed search strategies for other databases are provided in Supplementary Table 1 (available at: http://links.lww.com/JS9/D920).

### Inclusion and exclusion criteria

Studies were selected based on the following PICOS criteria, regardless of language: (1) population: all patients who underwent pancreaticoduodenectomy, regardless of tumor pathology (benign or malignant), histological type, and surgical approach (open, laparoscopic, or robotic pancreaticoduodenectomy); (2) intervention: assessment of risk factors for failure to achieve textbook outcome, including gender, age, BMI, malignant pathology, preoperative serum albumin level, preoperative biliary drainage, pancreatic texture, pancreatic duct diameter, portal vein resection, blood transfusion, blood loss, and operation time; (3) comparison: characteristics of these factors in patients achieving textbook outcome; (4) outcome: risk factors of failure to achieve textbook outcome after PD, expressed as odds ratios (ORs) with 95% confidence intervals (CIs); (5) study design: randomized controlled trials (RCTs) and observational studies, including case-control, cohort, and cross-sectional studies.

Exclusion criteria were: (1) studies including surgical procedures other than PD, (2) studies with incomplete data or unavailable original data, and (3) non-research articles (reviews, commentaries, case reports, etc.).

### Data extraction and quality assessment

After removing duplicates, two investigators independently conducted three-level screening of titles, abstracts, and full texts. A third investigator independently reviewed 10% of randomly selected samples, achieving 80% consistency in preliminary screening. Disagreements were resolved through discussion with a fourth investigator. Two investigators independently extracted the following information from each included study: first author, publication year, data collection period, study design, country, sample size, number of patients failing to achieve textbook outcome (non-TO), relevant factors, and independent predictors.The methodological quality of included studies was assessed using the Newcastle-Ottawa Scale (NOS)^[14]^ by two reviewers. The NOS evaluates three domains: selection (four points), comparability (two points), and outcome (three points), with a total score of nine points. Studies could be awarded a maximum of one point for each item within the selection and outcome categories, and a maximum of two points for comparability. Studies with ≥6 points were considered to have low risk of bias.

### Statistical analysis

Pooled odds ratios (ORs) with 95% confidence intervals (CIs) were calculated using the Mantel-Haenszel method. Heterogeneity among studies was assessed using the I^2^ statistic and Cochran’s Q test, with *P* <0.1 considered statistically significant. Heterogeneity was categorized as low (I^2^ < 25%), moderate (25% ≤ I^2^ ≤ 50%), or high (I^2^ > 50%). Random-effects models were applied when I^2^ > 50%; otherwise, fixed-effects models were used. Sensitivity analyses and subgroup analyses were conducted on selected outcomes to evaluate their influence on the pooled effect size. Publication bias was assessed using Harbord’s test in Stata software, with *P* >0.05 indicating no significant publication bias. All statistical analyses were conducted using Review Manager 5.4 and Stata 17 software, with *P* <0.05 considered statistically significant.

## Results

### Study selection

**Figure 1.**
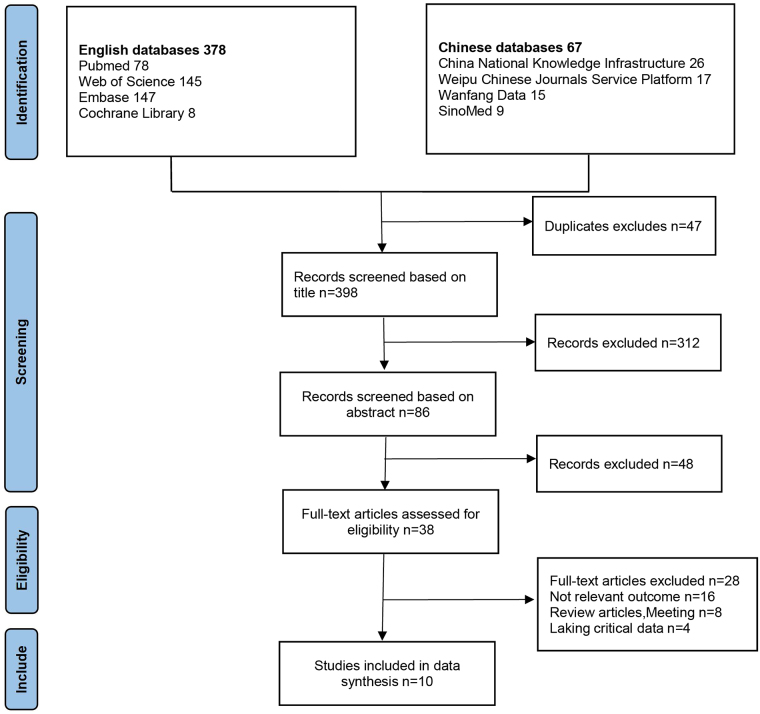
PRISMA (Preferred Reporting Items for Systematic Reviews and Meta-Analyses) flow diagram showing a selection of studies for review.

Of the initial 445 articles identified, 47 duplicates were removed, leaving 398 articles. After title screening, 312 irrelevant articles were excluded, leaving 86 articles. Abstract screening excluded 48 articles that did not meet the inclusion criteria, leaving 38 articles. Full-text review further excluded 28 articles that did not meet the inclusion criteria. Finally, 10 studies (7 in English and 3 in Chinese) involving 5238 patients were included^[5,10,11,15-21]^. The study selection process is detailed in Fig. 1.

**Figure 2.**
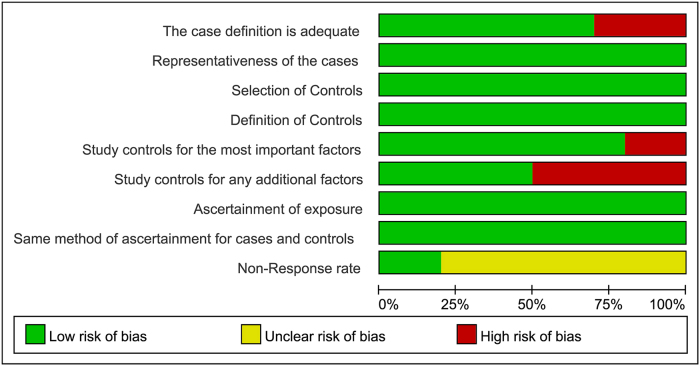
Risk of bias graph: review authors’ judgments about each risk of bias item presented as percentages across all included studies.

### Characteristics of included studies

The 10 included studies were published between 2020 and 2024, with sample sizes ranging from 85 to 2633 patients. These studies were primarily conducted in China, with a few from European countries. The incidence of failure to achieve textbook outcome after pancreatoduodenectomy ranged from 17.50% to 78.26%, with an overall rate of 44.29%. Detailed characteristics are presented in Table 1.

**Table 1:** Descriptive information of the included studies of risk factors for failure to achieve textbook outcome in patients after pancreaticoduodenectomy

Reference		Year	Data collection time		Study design	Country	Center	Surgical approach		Histological type	
Cai H *et al* ^[15]^		2024	2019.11-2022.11		Prospective	China	West China Hospital, Sichuan University	LPD		NA	
van Roessel S *et al* ^[10]^		2020	2014-2017		Prospective	Netherlands	The nationwide prospective Dutch Pancreatic Cancer Audit	OPD: 86.2%		NA	
								LPD or RPD: 13.8%			
Wang H *et al* ^[16]^		2024	2018.01-2021.12		Retrospective	China	Sun Yat-sen Memorial Hospital, Sun Yat-sen University	OPD: 52.4%		Pancreatic ductal adenocarcinoma	
								LPD: 40.5%			
								RPD: 7.1%			
Wu Y *et al* ^[17]^		2023	2010.01-2016.08		Retrospective	China	16 multi-centers (Supplementary Table 2, available at: http://links.lww.com/JS9/D921)	LPD		NA	
Zhang XJ *et al* ^[18]^		2023	1998-2020		Retrospective	China	The China National Cancer Center	NA		Ampullary carcinoma	
Partelli S *et al* ^[19]^		2024	2007-2017		Retrospective	Italy	The International multi-center	OPD: 97% LPD or RPD: 93%		Nonfunctioning pancreatic neuroendocrine tumors (NF-PanNETs)	
Yu Y *et al* ^[20]^		2024	2020.03-2023.05		Retrospective	China	China–Japan Union Hospital of Jilin University	RPD		Pancreatic cancer	
Tang CQ *et al* ^[5]^		2024	2019.01-2022.12		Retrospective	China	Henan University Peoples Hospital	OPD: 53.7% LPD or RPD: 46.3%		Pancreatic ductal adenocarcinoma	
Deng G *et al* ^[11]^		2023	2016.01-2018.12		Retrospective	China	The Second Affiliated Hospital of Kunming Medical University	NA		Pancreatic head carcinoma	
Hu ZX *et al* ^[21]^		2023	2017.12-2021.12		Retrospective	China	The Second Affiliated Hospital of Kunming Medical University	OPD: 73.9%		Periampullary cancer	
								LPD: 26.1%			
Reference	**Number of patients**			**Non-TO case**	**Influence factors investigated**				**Independent Influence factors**	
Cai H *et al* ^[15]^	200			35	Age, Sex, BMI, ASA score, APTT, PT, Hemoglobin, Albumin, Creatinine, Total bilirubin, Biliary drainage, Tumor size, OT, EBL, Blood transfusion, Time of PJ, MPD stent, Pancreatic texture, Diameter of MPD, Postoperative hospital stays, Diagnosis, Pathological outcomes				Sex, Pancreatic texture	
van Roessel S *et al* ^[10]^	2633			1097	Age, Sex, ASA score, BMI, Performance status, Dilated pancreatic duct, Neoadjuvant therapy, Histological diagnosis, Vascular resection				ASA III, Dilated pancreatic duct, PDAC	
Wang H *et al* ^[16]^	111			42	Gender, Age, BMI, ASA, Resectability, CA199, APTT, Neutrophils, Lymphocyte, Monocyte, NLR, DFA POD, Operation duration,Operation type, Pancreatic anastomosis, Estimated blood loss, Blood transfusion, TNM, Tumor size, Resected lymph nodes,Pancreatic texture, Pancreatic duct diameter, LOS, LOFAT, PATc, CPAT,				DFA POD, Operation duration, Pancreatic texture	
Wu Y *et al* ^[17]^	1029			320	Sex, Age, BMI, ASA, TBIL, Comorbidity, Pylorus-preserving LPD, Operative time, Pancreatic anastomosis, Removal of NGT during operation, Estimated blood loss, Number of transfusion, Transfusion, Dilated pancreatic duct, Texture of pancreas, Pathological outcomes, Tumor size, Number of lymph nodes, Postoperative hospital stays				Dilated pancreatic duct, Age, Cardiovascular disease	
Zhang XJ *et al* ^[18]^	272			206	Year of surgery, Sex, Age, Operation time, Blood transfusion, Tumor size, Differentiation, CA199, N stage, T stage, TNM stage, Lymphovascular invasion, Adjuvant treatment				Year of surgery, N stage	
Partelli S *et al* ^[19]^	477			326	Age, Sex, BMI, Tumor size, T stage, N Stage, M stage, Ki67 index, Lymphovascular invasion, Perineural invasion, Lymph node ratio≥0.12, Minimally invasive surgery, Venous resection, Intraoperative blood loss, Time of surgery, Surgical volume				Tumor size, Minimally invasive surgery, Surgical volume	
Yu Y *et al* ^[20]^	85			39	Sex, Age, Smoking history, Alcohol history, Weight, BMI, ASA score, Albumin, Total bilirubin, ALT, AST, PTCD, CA199, Abdominal operation history, Lymph node, Tumor size, Malignant tumor, Vascular invasion, Nerve invasion				None	
Tang CQ *et al* ^[5]^	205			92	Sex, Age, Length of stay, BMI,Albumin, Total Bilirubin, CA199,Tumor length, Lymph node metastases, Level of differentiation, Vascular tumor thrombus, Nerve invasion, Surgical method, Operation time, Intraoperative blood loss, Blood transfusion volume, CT radiation value, Pancreatic duct diameter				Level of differentiation, Operation time, Blood transfusion volume, CT radiation value	
Deng G *et al* ^[11]^	92			72	Sex, Age, Albumin, Total Bilirubin, CA199, Operation time, Intraoperative blood loss, TNM stage, N stage, Tumor length, Level of differentiation, Nerve invasion, Vascular invasion, Length of stay				Operation time,Intraoperative blood loss,TNM stage	
Hu ZX *et al* ^[21]^	134			91	Age, Sex, BMI, Long-term smoking history, Long-term drinking history, Hepatitis B history, Preoperative biliary drainage, Preoperative asymptomatic leukocytosis, Total Bilirubin, Albumin, CA199, CA125, CEA, Surgical method, Intraoperative blood loss, Operation time, Tumor length, Level of differentiation, Lymph node metastases, Nerve invasion, Vascular invasion, Radiotherapy and chemotherapy				Intraoperative blood loss, Lymph node metastases, Preoperative biliary drainage, Surgical method	

LPD, Laparoscopic Pancreaticoduodenectomy; RPD, Robotic Pancreaticoduodenectomy; OPD, Open Pancreaticoduodenectomy; NA, Not Available.

BMI, body mass index; ASA, American Society of Anesthesiologists classification; APTT, activated partial thromboplastin time; PT, prothrombin time; OT, operation time; EBL, estimated blood loss; PJ, Pancreaticojejunostomy; MPD, main pancreatic duct; NLR, neutrophil/lymphocyte ratio; DFA, drainage fluid amylase; POD, postoperative day; TNM (AJCC 8th), TNM stage of American Joint Committee on Cancer (eighth edition); LOS, length of postoperative hospital stay; LOFAT, length of time to the first adjuvant therapy; PATc, postoperative adjuvant therapy cycles; CPAT, completed postoperative adjuvant therapy. TBIL, total bilirubin. LPD, laparoscopic pancreaticoduodenectomy; NGT, nasogastric tube; ALT, Alanine aminotransferase; AST, aspartate aminotransferase; PTCD, percutaneous transhepatic cholangiodrainage; CA199, carbohydrate antigen199; CA125, carbohydrate antigen125.

### Quality assessment

The quality of included studies was evaluated using the Newcastle-Ottawa Scale (NOS) (see Table 2): only one study scored nine points, three studies scored eight points, three studies scored seven points, and three studies scored six points. All studies met or exceeded the quality threshold of six points, indicating overall high methodological quality of the included literature.

**Table 2:** Assessment of quality of included studies with the Newcastle–Ottawa Scale

	Component;score										
	Selection				Comparability		Exposure			Study quality	
Study	The case definition is adequate	Representativeness of the cases	Selection of Controls	Definition of Controls	Study controls for the most important factors	Study controls for any additional factors	Ascertainment of exposure	Same method of ascertainment for cases and controls	Non-response rate	Total score	Interpretation
Cai H *et al* ^[15]^	1	1	1	1	1	1	1	1	1	9	Good
van Roessel S *et al* ^[10]^	1	1	1	1	1	0	1	1	1	8	Good
Wang H *et al* ^[16]^	1	1	1	1	1	1	1	1	0	8	Good
Wu Y *et al* ^[17]^	1	1	1	1	1	1	1	1	0	8	Good
Zhang XJ *et al* ^[18]^	0	1	1	1	0	1	1	1	0	6	Good
Partelli S *et al* ^[19]^	0	1	1	1	1	0	1	1	0	6	Good
Yu Y *et al* ^[20]^	0	1	1	1	1	1	1	1	0	7	Good
Tang CQ *et al* ^[5]^	1	1	1	1	1	0	1	1	0	7	Good
Deng G *et al* ^[11]^	1	1	1	1	0	0	1	1	0	6	Good
Hu ZX *et al* ^[21]^	1	1	1	1	1	0	1	1	0	7	Good

**Figure 3.**
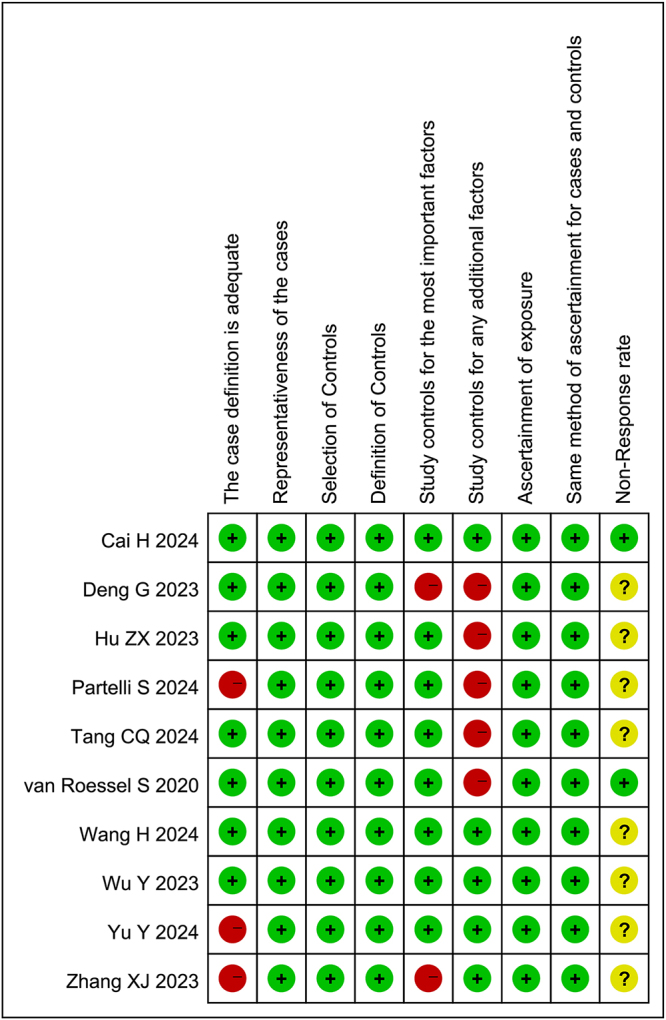
Risk of bias summary: review authors’ judgments about each risk of bias item for each included study.

Additionally, for each of the included studies, risk of bias graphs was produced. Each risk of bias item was presented as percentages across all included studies (Fig. 2). For individuals, a risk of bias summary was performed (Fig. 3). High risks of bias were seen for items of “the case definition is adequate,” “study controls for the most important factors” and “study controls for any additional factors.” Unclear risks of bias were observed only in items of “non-response rate.” Moreover, through meticulous examination of the original Chinese publications, we confirmed the absence of overlap in both research centers and data collection periods among the included studies (see Table 1 and Supplementary Table 2, available at: http://links.lww.com/JS9/D921).

### Meta-analysis results

**Figure 4.**
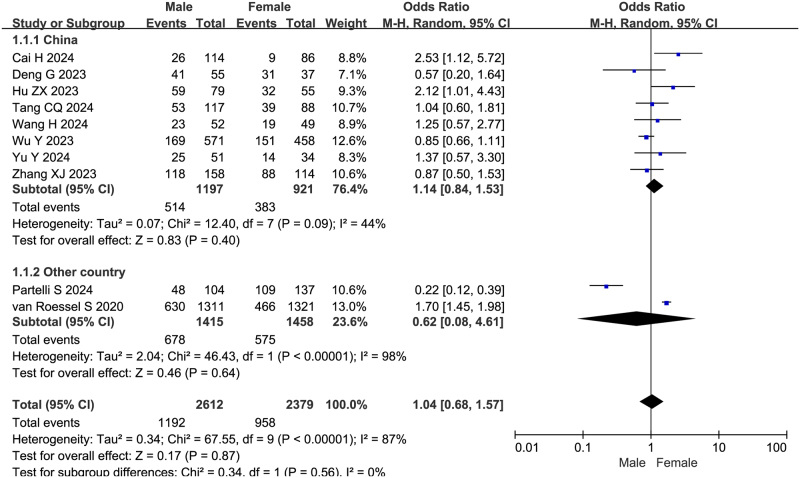
Forest plot of the association between gender and non-TO after pancreatoduodenectomy. df, degrees of freedom; M-H, Mantel-Haenszel.

This meta-analysis evaluated 18 potential risk factors (see Table 3), stratified into patient-related variables (gender, age, BMI, ASA score, preoperative albumin level, preoperative biliary drainage), tumor-related characteristics (tumor size, pancreatic texture, benign/malignant status, vascular invasion, lymph node metastasis, perineural invasion, histological differentiation, pancreatic duct diameter), and operative parameters (estimated blood loss, transfusion requirement, surgical approach, operative duration). Meta-analysis revealed that preoperative biliary drainage, smaller tumor size, soft pancreatic texture, small pancreatic duct diameter, and increased intraoperative blood loss were significantly associated with failure to achieve textbook outcome following pancreatoduodenectomy (*P* ≤ 0.05). The remaining 13 factors (detailed in Fig. 4 and Supplementary Figures, available at: http://links.lww.com/JS9/D919) demonstrated no significant correlation with textbook outcome achievement (*P* > 0.05).

**Table 3:** Summary of risk factors for failure to achieve textbook outcome after pancreatoduodenectomy

Characteristics	Factors	Number of articles	Participants	Statistical method	OR	95% CI	*P*	I^2^ (%)	PH
**Patient**	Sex	8	897	Odds ratio(M–H,Random,95%CI)	1.14	[0.84-1.53]	0.4	44%	0.09
	Age	6	3445	Odds ratio(M–H,Fixed,95%CI)	1.08	[0.91-1.27]	0.38	43%	0.12
	BMI	3	2947	Odds ratio(M–H,Fixed,95%CI)	1.15	[0.92-1.42]	0.22	33%	0.22
	ASA	4	3973	Odds ratio(M–H,Random,95%CI)	1.17	[0.83-1.65]	0.38	57%	0.07
	Albumin	3	431	Odds ratio(M–H,Random,95%CI)	1.01	[0.48-2.13]	0.97	59%	0.09
	Preoperative biliary drainage	3	419	Odds ratio(M–H,Fixed,95%CI)	2.09	[1.30-3.36]	0.002	37%	0.21
**Tumor**	Tumor Size	4	973	Odds ratio(M–H,Fixed,95%CI)	1.36	[1.02-1.81]	0.04	0	0.42
	Pancreas texture	3	1337	Odds ratio(M–H,Random,95%CI)	2.25	[1.01-5.02]	0.05	79%	0.09
	Diameter pancreatic duct	4	3978	Odds ratio(M–H,Random,95%CI)	2.30	[1.62-3.28]	<0.001	74%	0.009
	Malignant pathology	4	3947	Odds ratio(M–H,Fixed,95%CI)	0.91	[0.77-1.08]	0.3	0	0.46
	Vascular invasion	4	516	Odds ratio(M–H,Fixed,95%CI)	1.38	[0.91-2.09]	0.13	0	0.46
	Lymph node metastasis	6	1288	Odds ratio(M–H,Random,95%CI)	1.27	[0.72-2.23]	0.42	77%	0.0007
	Nerve invasion	5	993	Odds ratio(M–H,Fixed,95%CI)	1.08	[0.82-1.44]	0.58	6%	0.38
	Differentiation of tumor	4	703	Odds ratio(M–H,Random,95%CI)	1.42	[0.75-2.70]	0.28	54%	0.09
**Operation**	Blood transfusion	3	1501	Odds ratio(M–H,Fixed,95%CI)	1.15	[0.88-1.51]	0.31	0	0.57
	Estimated blood loss	4	542	Odds ratio(M–H,Random,95%CI)	4.14	[1.16-14.83]	0.03	83%	0.0006
	Minimally invasive surgery	4	3449	Odds ratio(M–H,Random,95%CI)	0.67	[0.30-1.52]	0.34	86%	<0.001
	Operation time	4	722	Odds ratio(M–H,Random,95%CI)	1.61	[0.67-3.90]	0.29	83%	0.0006

BMI, body mass index; ASA, American Society of Anesthesiologists classification; M–H, Mantel–Haenszel; OR, odds ratio.

### Gender

All 10 studies reported the association between gender and failure to achieve textbook outcome after pancreatoduodenectomy, with ORs ranging from 0.22 to 2.53. Nine studies showed no significant association, while only one study identified gender as an independent predictor. Heterogeneity testing of the 10 studies revealed significant heterogeneity (I^2^ = 87%, Q test *P* < 0.00001). After sensitivity and subgroup analyses, exclusion of two European studies (van Roessel S *et al* ^[10]^ and Partelli S *et al* ^[19]^) significantly reduced heterogeneity among the remaining eight Chinese studies (I^2^ = 44%, Q test *P* = 0.09). Meta-analysis using a random-effects model showed no significant association between gender and failure to achieve textbook outcome (OR = 1.14, 95%CI: 0.84-1.53, Z = 0.83, *P* = 0.40, PH = 0.09, I^2^ = 44%), as shown in Fig. 4.

### Preoperative biliary drainage

**Figure 5.**
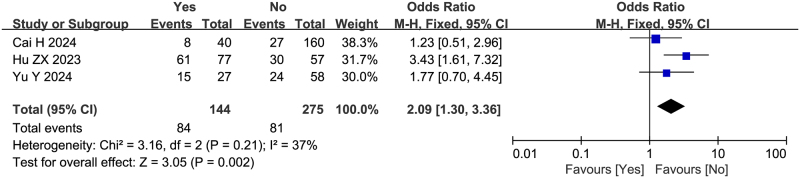
Forest plot of the association between preoperative biliary drainage and non-TO after pancreatoduodenectomy. df, degrees of freedom; M-H, Mantel-Haenszel.

Three studies (including 419 patients) reported the association between preoperative biliary drainage and failure to achieve textbook outcome after pancreatoduodenectomy, with ORs ranging from 1.77 to 3.43. Hu ZX *et al* identified preoperative biliary drainage as an independent risk factor^[21]^. Meta-analysis demonstrated a significant association between preoperative biliary drainage and failure to achieve textbook outcome (OR = 2.09, 95%CI: 1.30-3.36, Z = 3.05, *P* = 0.02, PH = 0.21, I^2^ = 37%), consistent with the findings of Hu ZX *et al*, as shown in Fig. 5.

### Tumor size

**Figure 6.**
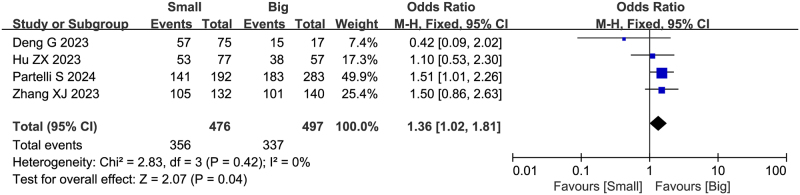
Forest plot of the association between tumor size and non-TO after pancreatoduodenectomy. df, degrees of freedom; M-H, Mantel-Haenszel.

Four studies (including 973 patients) reported the association between tumor size and failure to achieve textbook outcome after pancreatoduodenectomy. Partelli S *et al* ^[19]^ demonstrated that tumors >20 mm were more likely to achieve textbook outcome (OR = 1.702, 95%CI: 1.121-2.582, *P* = 0.013). Meta-analysis revealed that tumor size <20 mm was a risk factor for failure to achieve textbook outcome (OR = 1.36, 95%CI: 1.02-1.81, Z = 2.07, *P* = 0.04, PH = 0.42, I^2^ = 0%), indicating that smaller tumor size was significantly associated with failure to achieve textbook outcome, as shown in Fig. 6.

### Pancreatic texture

**Figure 7.**
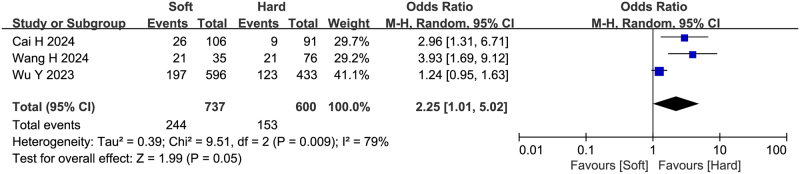
Forest plot of the association between pancreatic texture and non-TO after pancreatoduodenectomy. df, degrees of freedom; M-H, Mantel-Haenszel.

Three studies (including 1337 patients) reported the relationship between pancreatic texture and failure to achieve textbook outcome after pancreatoduodenectomy. The incidence of failure to achieve textbook outcome was 33.11% (244/737) in the soft texture group and 25.50% (153/600) in the firm texture group. Meta-analysis using a random-effects model demonstrated that soft pancreatic texture was a risk factor for failure to achieve textbook outcome (OR = 2.25, 95%CI: 1.01-5.02, Z = 1.99, *P* = 0.05, PH = 0.009, I^2^ = 79%). This finding was consistent with the results reported by Cai H *et al* ^[15]^ and Wang H *et al* ^[16]^, further confirming that patients with soft pancreatic texture were more likely to fail achieving textbook outcome. See Fig. 7 for detailed results.

### Pancreatic duct diameter

**Figure 8.**
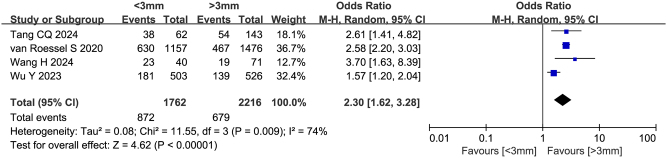
Forest plot of the association between pancreatic duct diameter and non-TO after pancreatoduodenectomy. df, degrees of freedom; M-H, Mantel-Haenszel.

Four studies (including 3978 patients) reported the association between main pancreatic duct diameter and failure to achieve textbook outcome after pancreatoduodenectomy, with ORs ranging from 1.57 to 3.70. Two studies^[10,17]^ identified pancreatic duct diameter as an independent predictor. Meta-analysis supported these findings (Fig. 8), demonstrating that pancreatic duct diameter <3 mm was a significant risk factor for failure to achieve textbook outcome (OR = 2.30, 95%CI: 1.62-3.28, Z = 4.62, *P* < 0.00001, PH = 0.009, I^2^ = 74%).

### Intraoperative blood loss

**Figure 9.**
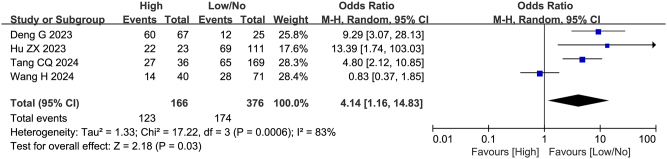
Forest plot of the association between intraoperative blood loss and non-TO after pancreatoduodenectomy. df, degrees of freedom; M-H, Mantel-Haenszel.

Four studies (including 542 patients) investigated the association between intraoperative blood loss and failure to achieve textbook outcome after pancreatoduodenectomy, with ORs ranging from 0.83 to 13.39. Two studies^[11,21]^ identified intraoperative blood loss as an independent predictor. Meta-analysis further supported these findings (Fig. 9), demonstrating that increased intraoperative blood loss was significantly associated with failure to achieve textbook outcome (OR = 4.14, 95%CI: 1.16-14.83, Z = 2.18, *P* = 0.03, PH = 0.0006, I^2^ = 83%), representing an important risk factor.

### Subgroup analysis

**Figure 10.**
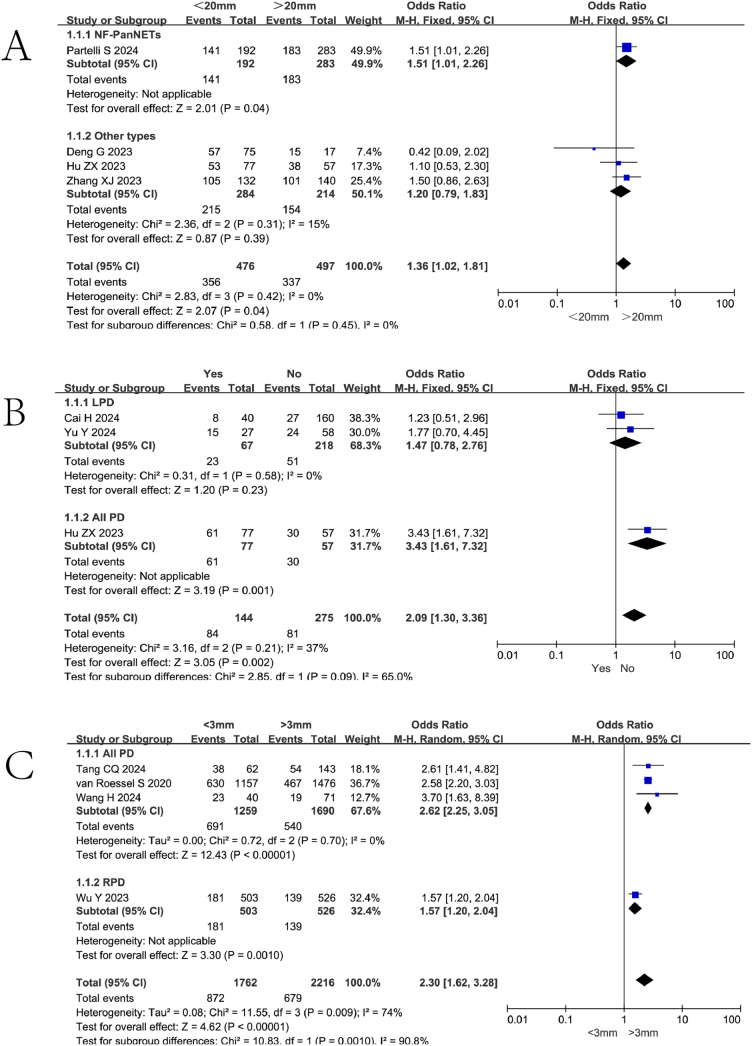
Subgroup analyses for various risk factors. df, degrees of freedom; M-H, Mantel-Haenszel.

Among the five risk factors analyzed, the Italian study^[19]^ focusing on non-functioning pancreatic neuroendocrine tumors (NF-PanNETs) was exclusively included in the “Tumor Size” analysis. Consequently, we performed a subgroup analysis stratified by tumor pathology for this specific risk factor (Fig. 10A). Notably, our subgroup analysis demonstrated that when the Italian study^[19]^ (NF-PanNETs) was excluded, tumor size <20 mm showed no significant association with failure to achieve textbook outcome (OR = 1.20, 95%CI: 0.79-1.83, Z = 0.87, *P* = 0.39, PH = 0.31, I^2^ = 15%).

With respect to surgical approaches, our included studies comprised one robotic pancreaticoduodenectomy (RPD) study and two laparoscopic pancreaticoduodenectomy (LPD) studies among the total ten studies. For the risk factors “Tumor Size” and “Intraoperative Blood Loss,” all studies were classified under “All PD” category (encompassing open, laparoscopic, and robotic approaches), thereby eliminating the necessity for subgroup analysis. Similarly, for “Pancreatic Texture,” the limited number of studies (one RPD, one LPD, and one All PD) precluded meaningful subgroup analysis.

Subgroup analysis of “Preoperative Biliary Drainage” (Fig. 10B) revealed that in LPD cases, preoperative biliary drainage demonstrated no significant correlation with failure to achieve textbook outcome (OR = 1.47, 95%CI: 0.78-2.76, Z = 1.20, *P* = 0.23, PH = 0.58, I^2^ = 0%). Furthermore, subgroup analysis of “Pancreatic Duct Diameter” (Fig. 10C) demonstrated that a pancreatic duct diameter <3 mm remained significantly associated with failure to achieve textbook outcome (OR = 2.62, 95%CI: 2.25-3.05, Z = 12.43, *P* < 0.00001, PH = 0.70, I^2^ = 0%), even after excluding the RPD study from the analysis.

### Publication bias

**Figure 11.**
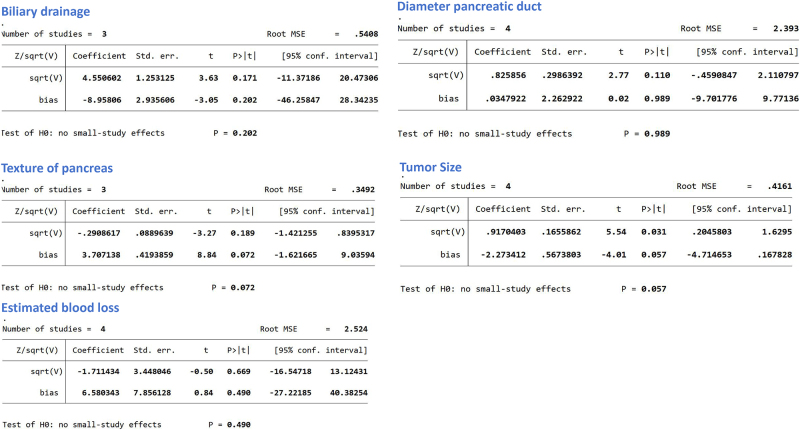
Harbord’s test for publication bias assessment. No significant publication bias was detected across all analyses (all *P* > 0.05).

Publication bias analysis was performed for studies investigating the five identified risk factors. The Harbord test was applied using Stata17 software for these dichotomous variables. The results showed no statistically significant publication bias for any of the risk factors (*P* > 0.05), indicating the absence of significant publication bias among the included studies, as shown in Fig. 11.

## Discussion

Pancreatoduodenectomy (PD), as one of the most technically demanding procedures in general surgery, cannot be adequately evaluated using single outcome measures. Textbook outcome (TO), a comprehensive multidimensional evaluation system, provides scientific evidence for objective quality assessment of PD and holds significant importance in promoting surgical standardization and optimization^[5]^. TO not only enables systematic evaluation and comparison of surgical quality across different institutions but also offers reliable guidance for patients’ medical decision-making. The application of TO has expanded from its origins in colorectal surgery to various surgical fields, including esophageal cancer, gastric cancer, organ transplantation, hepatobiliary tumors, and pancreatic cancer^[6,7,22-25]^, with rapidly growing research interest in PD. This meta-analysis systematically included all 10 studies since TO was first applied to PD in 2020, revealing that 44.3% of patients failed to achieve textbook outcome. Through systematic analysis of 18 potential risk factors, five significant predictors were identified: preoperative biliary drainage, smaller tumor size, soft pancreatic texture, small pancreatic duct diameter, and increased intraoperative blood loss. These findings provide an important theoretical foundation for preventing and reducing the failure to achieve textbook outcome in clinical practice.

Our meta-analysis identified preoperative biliary drainage as a significant risk factor for failure to achieve textbook outcome after pancreatoduodenectomy, consistent with the findings of Hu ZX *et al* ^[21]^. A multicenter randomized controlled trial^[26]^ demonstrated that preoperative biliary drainage significantly increased the incidence of serious surgical complications, with 39% (37 patients) in the non-drainage group versus 74% (75 patients) in the drainage group (relative risk in the non-drainage group, 0.54; 95% CI, 0.41 to 0.71; *P* < 0.001). However, subgroup analysis of “Preoperative Biliary Drainage” revealed no statistically significant increase in non-TO rates for LPD cases with preoperative biliary drainage. Upon reviewing the original articles, we found that both LPD studies exclusively utilized percutaneous transhepatic biliary drainage (PTBD), while the All PD study did not specify the drainage method. Previous study have demonstrated that PTBD, compared to ERCP with stenting, causes less local inflammation and consequently fewer adverse surgical outcomes^[27]^. Given the limited number of included studies, these pooled results should be interpreted with caution.

Partelli S *et al* ^[19]^ reported that tumor size >20 mm was positively associated with achieving textbook outcome after pancreatoduodenectomy in nonfunctioning pancreatic neuroendocrine tumors(NF-PanNETs), while tumor size <20 mm demonstrated no significant correlation. Our initial meta-analysis, incorporating data from four studies, suggested that smaller tumor size (<20 mm) was a risk factor for failure to achieve textbook outcome. However, subsequent subgroup analysis stratified by tumor pathology revealed that after excluding the Partelli S *et al* ^[19]^ study (NF-PanNETs), tumor size <20 mm was no longer identified as a risk factor for non-TO. This discrepancy might be explained by the underlying pancreatic parenchymal characteristics. In patients undergoing pancreatoduodenectomy, small tumor size (<20 mm) is frequently associated with normal pancreatic parenchyma, minimal inflammatory changes, and soft pancreatic texture. Furthermore, these small tumors typically exert less compression on the pancreatic duct, resulting in minimal ductal dilation^[28]^. Additionally, the impact of tumor size on TO may be influenced by tumor location; notably, tumors in the pancreatic tail rarely affect pancreatic duct diameter, as demonstrated by anatomical and imaging studies^[29]^. However, the current included studies do not support more detailed subgroup analyses based on tumor location. Therefore, the statistical findings regarding “tumor size” should be interpreted with caution, as they may be confounded by other variables, particularly “Pancreatic Texture” and “Pancreatic Duct Diameter,” suggesting that tumor size might not represent an independent risk factor. This conclusion warrants further validation through more detailed analyses considering both tumor location and pathological classification in future studies.

The International Study Group of Pancreatic Surgery (ISGPS) reported that soft pancreatic texture and pancreatic duct diameter <3 mm are significant risk factors for postoperative pancreatic fistula (POPF)^[30]^. Van Roessel S *et al* ^[10]^ and Wu Y *et al* ^[17]^ confirmed main pancreatic duct diameter as an independent predictor of failure to achieve textbook outcome after pancreatoduodenectomy, while Cai H *et al* ^[15]^ and Wang H *et al* ^[16]^ found pancreatic texture significantly associated with this outcome. Our meta-analysis further validated the importance of these two factors, demonstrating that both soft pancreatic texture and small pancreatic duct diameter significantly increase the risk of failure to achieve textbook outcome. The underlying mechanism may be that these factors increase the technical difficulty of pancreaticojejunostomy and elevate the risk of postoperative pancreatic fistula, thereby reducing the probability of achieving textbook outcome.

Our meta-analysis identified intraoperative blood loss as a crucial surgery-related factor affecting textbook outcome after pancreatoduodenectomy. The data revealed that patients with higher intraoperative blood loss had a significantly higher rate of failure to achieve textbook outcome (74.1%) compared to those with lower blood loss (46.3%). Increased intraoperative blood loss, often associated with vascular dissection, not only reflects surgical complexity but may also indicate inadequate hemostasis^[31]^. The main causes of significant intraoperative blood loss during pancreaticoduodenectomy can be classified into three aspects. First, anatomical variations, particularly hepatic arterial variants (such as right hepatic artery originating from SMA) and portal venous system variations, significantly increase the surgical difficulty and bleeding risk^[32]^. Second, vascular invasion by tumors, especially involvement of the portal vein-superior mesenteric vein (PV-SMV) axis, often results in vessel wall thinning and fragility, leading to increased risk of intraoperative hemorrhage. A meta-analysis of pancreatic resections^[33]^ demonstrated that patients undergoing PV-SMV resection had higher postoperative mortality compared to standard procedures (risk difference [RD] 0.01, 95%CI [0.00 to 0.03]; *P* = 0.2), with excessive blood loss being one of the main contributing factors. Third, chronic inflammation, such as that caused by preoperative ERCP-guided biliary stenting or pre-existing pancreatitis, results in perivascular inflammatory changes and fibrosis, making vascular dissection more challenging^[26]^.

Our meta-analysis found no significant association between 13 factors and failure to achieve textbook outcome after pancreatoduodenectomy. However, the influence of certain factors (such as operative time, tumor differentiation, and vascular invasion) requires further validation due to several study limitations. Firstly, the concept of Textbook Outcome (TO) was only recently established and implemented in pancreatic surgery in 2020. Despite our comprehensive database search, we identified a limited number of eligible studies, with some failing to strictly adhere to the DPCG’s standardized TO definition, and consequently, only 3–4 studies were included in the pooled analysis for each risk factor. Second, our subgroup analyses of five risk factors revealed significant heterogeneity in the pooled results: tumor type (nonfunctioning pancreatic neuroendocrine tumors) substantially influenced the “Tumor size” analysis, while surgical approach significantly affected the “preoperative biliary drainage” outcomes. Third, there was geographical imbalance in study distribution, with a predominance of Chinese studies and limited data from other regions. Fourth, regarding study design, only two studies employed a prospective approach, potentially introducing various forms of bias (information, selection, and recall bias), which may compromise the reliability of our findings. Finally, several important surgery-related factors could not be included due to insufficient data, such as internal versus external pancreatic drainage methods, reinforcement of gastroenterostomy, Heidelberg lymph node dissection protocol, and R0 resection details^[34]^. Considering these limitations, future large-scale, standardized, prospective multicenter studies are needed to further clarify risk factors for failure to achieve textbook outcome after pancreatoduodenectomy. We plan to update this meta-analysis when such high-quality evidence becomes available.

In conclusion, this meta-analysis systematically evaluated risk factors for failure to achieve textbook outcome after pancreatoduodenectomy and identified five significant predictors: preoperative biliary drainage, smaller tumor size, soft pancreatic texture, small pancreatic duct diameter, and increased intraoperative blood loss. As a multidimensional comprehensive evaluation indicator, textbook outcome not only reflects the overall quality of perioperative care but also serves as a reliable tool for prognostic assessment. These findings provide important reference for surgeons in preoperative risk assessment and help develop individualized surgical strategies.With the continuous advancement of healthcare quality management, incorporating textbook outcome into hospital surgical quality control systems has become an inevitable trend^[35]^. Future prospective studies focusing on surgical details are needed to further optimize pancreatoduodenectomy procedures, improve textbook outcome achievement rates, and ultimately achieve continuous improvement in surgical quality.

SDC Figs: http://links.lww.com/JS9/D919

## Data Availability

Any datasets generated during and/or analyzed during the current study are publicly available.

## Research registration unique identifying number (UIN)

This systematic review and meta-analysis was registered in PROSPERO (International Prospective Register of Systematic Reviews) with registration number CRD42024584672.
